# The Impact of a Fat-Dominant Preload Before a Carbohydrate-Rich Meal on Glucose Homeostasis in Patients Without Diabetes After Sleeve Gastrectomy: A Proof-of-Concept, Randomised, Open-Label, Crossover Study

**DOI:** 10.3390/nu18030469

**Published:** 2026-01-31

**Authors:** Gráinne Whelehan, Louisa Y. Herring, Aikaterina Tziannou, Joseph Henson, Alice E. Thackray, David Bowrey, Jenny Abraham, Vinod Menon, Iskandar Idris, Helen Waller, David J. Stensel, David R. Webb, Thomas Yates, Melanie J. Davies, Dimitris Papamargaritis

**Affiliations:** 1 Diabetes Research Centre, University of Leicester, Leicester LE1 7RH, UK; 2 Leicester Diabetes Centre, University Hospitals of Leicester NHS Trust, Leicester LE5 4PW, UK; 3 NIHR Leicester Biomedical Research Centre, Leicester General Hospital, Leicester LE5 4PW, UK; 4 National Centre for Sport and Exercise Medicine, School of Sport, Exercise and Health Sciences, Loughborough University, Loughborough LE11 3TU, UK; 5 Department of Surgery, University Hospitals of Leicester NHS Trust, Leicester LE1 5WW, UK; 6 Department of General Surgery, University Hospitals Coventry & Warwickshire NHS Trust Coventry, Coventry CV2 2DX, UK; 7 Warwick Medical School, University of Warwick, Coventry CV4 7AL, UK; 8 Faculty of Sport Sciences, Waseda University, Tokorozawa 359-1192, Japan; 9 Department of Sports Science and Physical Education, The Chinese University of Hong Kong, Ma Liu Shui, Hong Kong; 10 Department of Diabetes and Endocrinology, Kettering General Hospital NHS Trust, Kettering NN16 8UZ, UK

**Keywords:** bariatric surgery, sleeve gastrectomy, preload, fat-dominant, glucose, insulin, glucose homeostasis, c-peptide, glucagon-like peptide-1

## Abstract

Background/Objectives: Sleeve gastrectomy (SG) improves glycaemic control; however, it also markedly accelerates gastric emptying, which can lead to exaggerated postprandial glucose and insulin excursions and, in some cases, postprandial hyperinsulinaemic hypoglycaemia. In non-surgical populations, fat preloads can reduce postprandial glycaemia by slowing gastric emptying, but their effects after SG are unclear. Methods: Ten adults >1-year post-SG completed a randomised, open-label, crossover study involving two mixed-meal tolerance tests (MMTTs), preceded (−30 min) by either a moderate, fat-dominant preload (28 g Brazil nuts) or 100 mL water (control). Blood samples were collected over three hours to assess plasma glucose, insulin, c-peptide, and total glucagon-like peptide-1 (GLP-1). Hypoglycaemia and dumping symptoms were assessed using validated questionnaires. Nadir plasma glucose concentration was the primary outcome. Results: Nadir plasma glucose was identical between conditions (both 3.8 mmol/L; 95% CI: −0.4, 0.3, *p* = 0.849), and neither peak glucose nor overall postprandial glucose exposure (incremental area under the curve iAUC_0–180 min_) differed between the preload and water conditions. Insulin and c-peptide concentrations immediately before the MMTT were higher after the fat-dominant preload (both *p* < 0.001). Overall insulin and c-peptide responses during the MMTT (iAUC_0–180 min_) remained comparable between conditions (95% CI −225, 2665; *p* = 0.442 and −67,787, 70,263; 0.968), but peak values for both hormones were higher after the preload (95% CI 2.9, 79.1, *p* = 0.038 and 17.3, 2402.7, *p* = 0.040, respectively). Total GLP-1 was also elevated prior to the MMTT (95% CI 1.6, 22.8, *p* = 0.028), while its early and overall postprandial responses did not differ (both *p* > 0.05). Ratings of hypoglycaemia and dumping symptoms were similar for both study arms. Discussion: A moderate, fat-dominant preload consumed before a mixed meal did not alter nadir or overall postprandial glucose in adults without diabetes after SG. However, the preload was associated with higher peak insulin secretion, a finding that should be interpreted with caution, as the study was not powered for secondary outcomes. Given the unique gastrointestinal physiology after SG, further research is needed to determine how different nutrient compositions or timing approaches influence postprandial glucose homeostasis in this population.

## 1. Introduction

Sleeve gastrectomy (SG), the most commonly performed bariatric procedure worldwide, removes 70–80% of the stomach to create a narrow tubular gastric remnant [1]. This operation not only restricts stomach volume but also induces major physiological changes in nutrient handling. The tubular stomach and the loss of the fundic reservoir capacity lead to accelerated gastric emptying and rapid delivery of nutrients to the small intestine [2]. These changes appear to play an important role in improved glycaemic control and in the remission of early type 2 diabetes (T2D), commonly observed after SG [3]. However, these changes also increase glycaemic variability, characterised by relatively sharp postprandial glucose and insulin excursions compared with individuals who have not undergone bariatric surgery, which in some cases can lead to postprandial hyperinsulinaemic hypoglycaemia (PHH) [4,5].

Emerging evidence indicates that pre-meal ingestion of non-carbohydrate macronutrients (20–30 min before eating) can substantially attenuate postprandial glycaemia in individuals with and without T2D [6]. Protein preloads, particularly those containing whey protein, reduce glucose excursions by approximately 30–40% during oral glucose tolerance tests (OGTT) or mixed-meal tests through potent insulinotropic effects, enhancing early glucose-stimulated insulin secretion two- to three-fold [7,8,9]. In contrast, fat preloads primarily act by slowing gastric emptying and delaying glucose absorption [10,11], resulting in lower early insulin concentration and a delayed (rather than amplified) insulin peak. Mixed preloads combining protein and fat appear to provide additive benefits (stimulating early insulin and incretin release while slowing gastric emptying), yielding up to 30–50% reductions in postprandial glucose excursions and improving β-cell glucose sensitivity [12,13]. In real-world settings, when individuals choose a snack that is virtually carbohydrate-free, it is typically a combination of fat and protein rather than exclusively one macronutrient. However, most preload studies to date have used protein-dominant mixtures, which may not be optimal after bariatric surgery given that robust protein-stimulated insulin secretion could increase the risk of PHH.

Following SG, although gastric emptying is accelerated, a functional gastric remnant remains—unlike in Roux-en-Y gastric bypass (RYGB), where only a small pouch is retained [14]. In this context, a fat-dominant, virtually carbohydrate-free preload with moderate energy and protein content may offer a physiologically appropriate strategy to slow the rate of gastric emptying, modulate exaggerated early insulin secretion, and support more stable postprandial glucose levels. This study aimed to test whether consuming a fat-dominant preload (Brazil nuts) 30 min before a carbohydrate-rich mixed-meal would alter the post-meal plasma glucose and insulin responses in adults without diabetes after SG. Accordingly, the focus was on metabolic responses, rather than on demonstrating mechanisms related to gastric emptying. Brazil nuts were selected to represent a realistic, virtually carbohydrate-free snack commonly consumed after SG, while a mixed-meal test was chosen as a physiologically appropriate postprandial stimulus that reduces the risk of dumping symptoms compared with an OGTT. We hypothesised that the preload would be associated with postprandial metabolic changes, potentially consistent with slower nutrient passage to the small intestine, leading to a more gradual glycaemic response, a lower postprandial insulin peak, and a higher plasma glucose nadir concentration.

## 2. Methods

### 2.1. Participants

Ten adults (aged 18–65 years) without diabetes who had undergone sleeve gastrectomy (SG) more than one year prior were recruited. Key exclusion criteria included established diagnosis of PHH, on treatment with medications that can affect glucose homeostasis (such as glucose-lowering agents or systemic steroids), history of any bariatric procedure other than SG, and the presence of diabetes. Full inclusion and exclusion criteria are provided in the Supplementary Material.

This study was approved by the Yorkshire & The Humber—Bradford Leeds Research Ethics Committee (REC ref: 20/YH/0339, date of approval 26 January 2021). The trial was prospectively registered on the ISRCTN Registry (ISRCTN16600471, date of registration 19 March 2021). All participants provided written informed consent before participation in the study. Data collection was completed between May 2022 and October 2023.

### 2.2. Study Design

**Figure 1:**
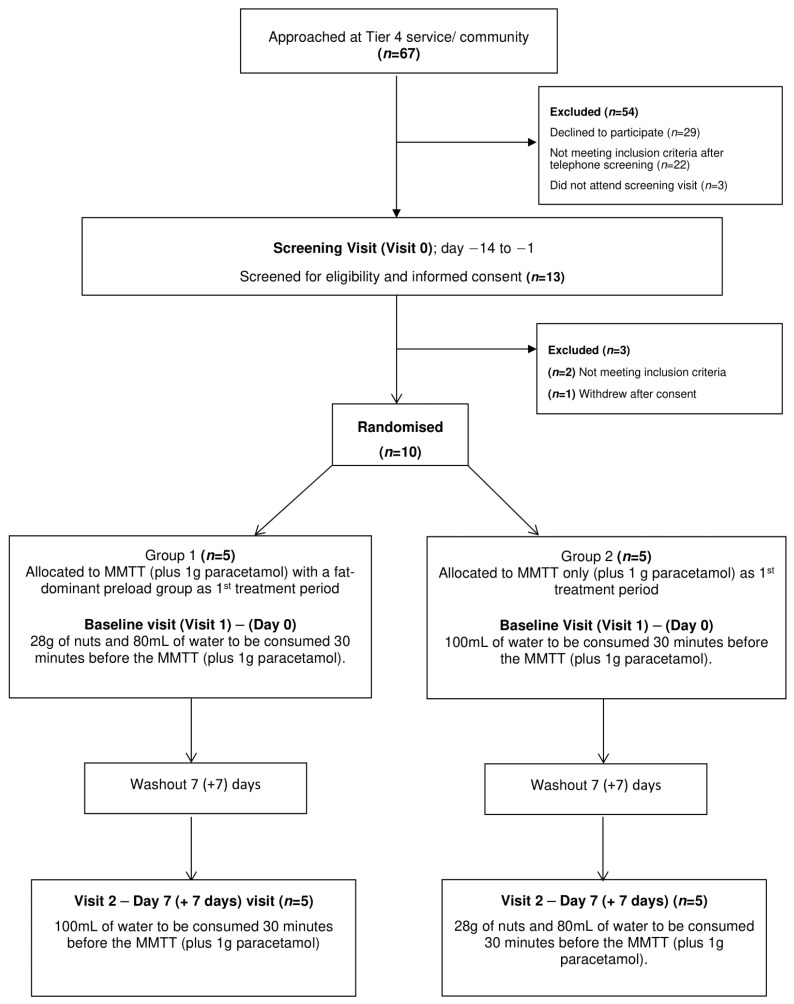
Flowchart of study procedures.

This was a proof-of-concept, randomised, open-label, crossover study (Figure 1). All study visits took place at the Leicester Diabetes Centre, UK. Participants attended an initial screening visit followed by two experimental visits which were separated by a washout period of 7 days. On each experimental visit, participants underwent a standardised mixed-meal tolerance test (MMTT) [15]. Participants were randomised using an online computerised randomisation service (sealed.envelope.com) to one of the two sequences: receiving either a fat-dominant preload (with 80 mL of water) or 100 mL of water alone, 30 min before the MMTT on their first visit. Participants then received the alternate condition on the subsequent experimental visit.

During the screening visit a general physical examination was performed by a trained delegated clinician. Blood samples were collected to assess HbA1c, full blood count, renal function, and liver function. All female participants of child-bearing potential had a urine pregnancy test to confirm their pregnancy status. Demographic and medical history information was also collected. After confirmed eligibility, participants attended the first experimental visit.

### 2.3. Mixed-Meal Tolerance Test (MMTT)

In the 24 h preceding the MMTT visits, the participants were asked to refrain from completing any moderate to vigorous physical activity and from consuming any alcohol. Forty-eight hours before the MMTT participants were asked to refrain from consuming paracetamol in order to avoid drug toxicity (as paracetamol was administered as part of the intervention as an intended marker of gastric emptying). On the morning of each experimental visit, participants arrived fasted (from 11 p.m. the evening prior) at the Leicester Diabetes Centre. Body weight, body fat percentage, and muscle mass were recorded using bioelectrical impedance equipment (Tanita^TM^ scales [Tanita, West Drayton, UK]). Blood pressure and resting heart rate were then measured using an automated sphygmomanometer for the arm whilst the participant was seated after resting quietly for five minutes. A cannula was then inserted into the antecubital fossa vein for repeated blood sampling. A fasting blood sample was taken (at −30 min before MMTT) before the fat-dominant preload (28 g Brazil nuts with 80 mL water) or 100 mL water was consumed. In total, 28 g of Brazil nuts contains 201 kcals, 19.5 g fat (4.5 g saturated fat, 5.7 g monounsaturated fat, 8.5 g polyunsaturated fat), 0.8 g carbohydrate, and 4.8 g protein. The preload and water, or water alone, were consumed over 10 min. Blood samples were collected at −30, −15, and immediately before the MMTT (0 min) and 15, 30, 45, 60, 90, 120, 150 and, 180 min after MMTT ingestion. The MMTT comprised 220 mL Nutricia Fortisip Milkshake (330 kcals, 12.8 g fat, 40.5 g carbohydrates, 13.2 g protein) and 1 g dispensable paracetamol (used as a test for gastric emptying). For safety reasons, bedside blood glucose was also performed at the same time points as blood collection and if blood glucose levels were ≤3 mmol/L, then participants were treated as per the “Hypoglycaemia Guideline for Adults” by the University Hospitals of Leicester and the MMTT was stopped. The MMTT was stopped for one participant at 60 min post-MMTT ingestion (during preload condition) for this reason.

### 2.4. Questionnaires

The Edinburgh Hypoglycaemia Scale (EHS) [16] and the Sigstad dumping score [17] questionnaires were provided to participants to complete both prior to and after the MMTT, immediately after each blood draw. The EHS assessed symptoms of hypoglycaemia during the MMTT, and the Sigstad assessed dumping symptoms.

### 2.5. Analytical Procedures

Whole blood was dispensed for measurement of plasma glucose into 2.7 mL fluoride/oxalate tubes immediately after collection. Tubes were then transferred to the main diagnostic laboratory of the University Hospitals of Leicester for the measurement of plasma glucose using a Glucose Hexokinase 3 assay on the Atelica CH (SIEMENS, Munich, Germany) analyser.

Blood samples for insulin and c-peptide were collected into pre-cooled Ethylenediaminetetraacetic acid tubes (EDTA 4.9 mL). Blood samples for total GLP-1 analysis were collected into EDTA tubes pre-treated with 250 µL aprotinin to ensure sample viability for analysis. All samples were immediately centrifuged at 4 °C and 1500× *g* for 10 min. Plasma was then aliquoted into 1 mL Eppendorf tubes and frozen, initially at −20 °C then transferred for storage at −80 °C on the same day as collection. Samples for total GLP-1 analysis were transported by dry ice and analysed by Loughborough University using a commercially available enzyme-linked immunosorbent assay (Merck KGaA, Darmstadt, Germany). Insulin and c-peptide were analysed at the Leicester Diabetes Centre using commercially available multiplex assays (Biotechne, Minneapolis, MN, USA). Total GLP-1 assay CV was 3.2%, insulin and c-peptide were 4.0 and 2.7%, respectively. All samples were handled in accordance with Human Tissue Authority’s Code of Practice.

For the assessment of serum paracetamol, whole blood was dispensed into serum-separating tubes immediately after collection. Samples were then transferred to the University Hospitals of Leicester laboratories for analysis.

### 2.6. Statistical Analysis

The primary outcome was the difference in nadir plasma glucose concentration after the standardised MMTT with or without the fat-dominant preload. This was a proof-of-concept study, and there is a lack of available data on the effects of fat preloads on glucose homeostasis after SG. However, based on previous literature [18,19], and assuming a standard paired design with α = 0.05, a within-person correlation of 0.05, and a standard deviation of 0.40 mmol/L, a sample size of 10 participants would provide 80% power to detect a 0.6 mmol/L difference in nadir plasma glucose between conditions. Secondary outcomes addressed secondary objectives of characterising the accompanying postprandial metabolic and hormonal responses following the preload intervention and included differences in fasting and pre-MMTT levels (immediately before MMTT), as well as overall (0–180 min) and early (0–30 min) post-MMTT incremental area under the curve (iAUC) for glucose, insulin, GLP-1, c-peptide, and paracetamol. Peak was defined as the highest observed post-MMTT concentration of each parameter for each participant. The trapezoidal rule was used to calculate iAUC. Paired *t*-tests or Wilcoxon signed-rank tests were used to analyse the primary and secondary outcomes on a complete case basis. Effect size was reported as Cohens d for all outcomes. Normality of all data was assessed using histograms and confirmed by the Shapiro–Wilk test. Depending on the distribution of data, participant characteristics were reported as mean (SD) or median (IQR), and number (percentage) for continuous and categorical variables, respectively. For the ten participants included in the analysis, 2.7% of plasma values were missing (6/220 values) and imputed using a regression method reported previously for acute experimental studies [20]. This model used key predictors (age, baseline BMI, fasting values) to derive a regression equation for the glucose, insulin, c-peptide, and GLP-1 values at each missing time point. A value of *p* < 0.05 was considered statistically significant for all analyses. Secondary outcomes were analysed as exploratory endpoints, and no adjustment for multiple comparisons was applied. Data were interpreted considering the *p*-value, effect size, and overall pattern of results. The analyses were conducted using R software version 4.3.1 and SPSS (version 29.0).

## 3. Results

### 3.1. Study Population

Ten adults (nine female/one male [broadly consistent with the typical bariatric surgery population]) who have previously undergone SG took part in the study. Participant characteristics are shown in Table 1.

### 3.2. Plasma Glucose

**Figure 2:**
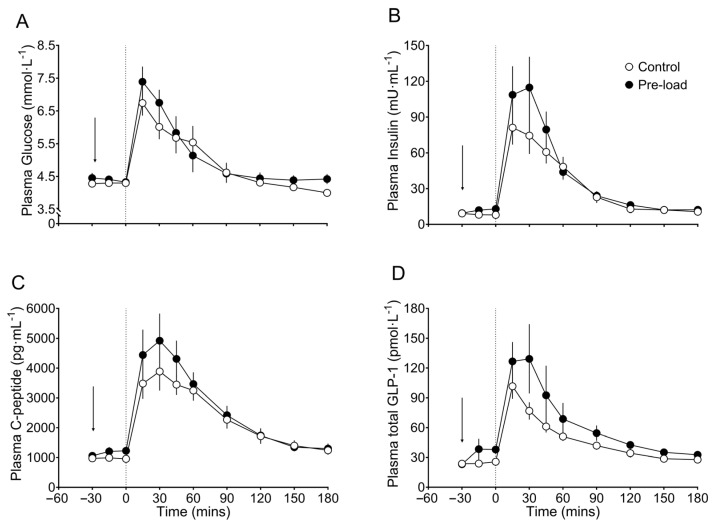
Postprandial time-course data of postprandial metabolites. Plasma glucose (**A**), plasma insulin (**B**), plasma c-peptide (**C**) and plasma total GLP-1 (**D**) are shown. Data depicts mean using white circles for the control (water) condition and black circles for the treatment (fat-dominant preload) condition. Error bars represent standard error. Dashed line at timepoint 0 represents time at which the MMTT was administered. The arrow depicts the timepoint at which the fat-dominant preload or the control condition was administered.

The time-course of plasma glucose response is shown in Figure 2A. The primary outcome, mean glucose nadir concentration, did not differ between treatments (3.8 [0.5] vs. 3.8 [0.7] mmol/L, for water and preload, respectively *p* = 0.849) Table 2.

Peak glucose during the MMTT was 7.2 ± 1.1 and 7.8 ± 1.4 mmol/L after water and preload (*p* = 0.164), respectively. Peak to nadir glucose concentration and pre-MMTT plasma glucose concentration were also similar between conditions (*p* > 0.05). Fasting plasma glucose did not differ between the two conditions (4.6 [0.5] mmol/L with the preload versus 4.3 ± 0.3 mmol/L with the water, *p* = 0.071, Table 2). Early post-MMTT glucose iAUC_0–30 min_ was not statistically different between the fat-dominant preload and water (64 ± 26 versus 49 ± 20 mmol∙min/L, respectively, *p* = 0.080).

### 3.3. Plasma Insulin

Plasma insulin concentration was similar at fasting (−30 min) prior to the ingestion of the preload (9.4 ± 5.8 µU/mL) or water (9.2 ± 5.6 µU/mL), *p* = 0.835, Table 2. Immediately before the MMTT, the fat-dominant preload increased the plasma insulin concentration compared with water (12.9 ± 6.7 vs. 8.0 ± 4.7 µU/mL, *p* < 0.001).

During the MMTT, the fat-dominant preload resulted also in a higher peak insulin concentration (134.0 ± 88.1 vs. 93.1 ± 46.2 µU/mL, *p* = 0.038, Figure 2B). Meanwhile, overall (0–180 min) and early (0–30 min) insulin iAUC were not statistically different between the preload and water conditions, with values of 5036 ± 2883 versus 4560 ± 2202 µU∙min/mL (*p* = 0.422), and 2200 ± 1617 versus 1596 ± 941 µU∙min/mL (*p* = 0.077), respectively (Table 2).

### 3.4. Plasma c-Peptide

Plasma c-peptide concentration mirrored the plasma insulin response (Figure 2C). Fasting c-peptide levels were similar between the preload and water conditions (1057 ± 311 vs. 974 ± 375 pg/mL, *p* = 0.111). Immediately before the MMTT, however, c-peptide was higher after the preload (1226 ± 382 vs. 957 ± 337 pg/mL, *p* < 0.001).

During the post-MMTT period, the preload also produced a higher peak c-peptide concentration (5542 ± 3049 vs. 4332 ± 1797 pg/mL, *p* = 0.040). Meanwhile, overall postprandial c-peptide iAUC_0–180 min_ did not differ between conditions (*p* = 0.968), and the early c-peptide response (iAUC_0–30 min_) also did not reach statistical significance compared with water (*p* = 0.112) (Table 2).

### 3.5. Plasma Total GLP-1

Time-course of plasma total GLP-1 response is shown in Figure 2D. There was no difference in fasting plasma total GLP-1 concentration before ingestion of either the preload or water (23.2 ± 8.8 vs. 23.7 ± 10.4 pmol/L, *p* = 0.876). However, after ingestion of the fat preload, plasma total GLP-1 concentration increased by 64% (to 38.0 ± 9.8 pmol/L), whereas levels remained at 25.7 ± 10.3 pmol/L after water (11% increase), resulting in a significant difference immediately before the MMTT (*p* = 0.028).

After MMTT ingestion, peak postprandial total GLP-1 concentrations did not differ between conditions (114.4 ± 108.0 vs. 94.8 ± 37.1 pmol/L, *p* = 0.232), although the timing of peak varied, occurring at 30 min after the preload and at 15 min after water. Early GLP-1 response (iAUC_0–30 min_) was not statistically different between conditions (*p* = 0.403) and the overall GLP-1 iAUC_0–180 min_ also showed no difference (*p* = 0.581).

### 3.6. Gastric Emptying

Paracetamol concentrations were reported by the UHL clinical laboratory, which did not quantify values below 10 mg/L due to lack of validated in-house precision at this level. As a result, many values were reported as “<10”, preventing reconstruction of a complete time-course profile. Consequently, these data could not be used to assess gastric emptying.

### 3.7. Questionnaires

**Figure 3:**
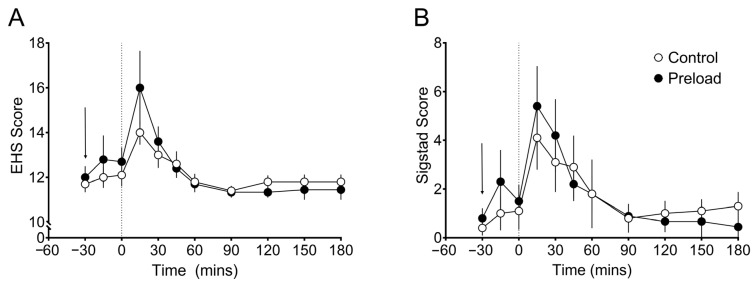
Dumping and hypoglyaemic scores. Data shows mean and standard error from the Edinburgh scale (**A**) and Sigstad score (**B**). Dashed line at timepoint 0 represents time at which the MMTT was administered. The arrow depicts the timepoint at which the fat-dominant preload or the control condition was administered.

There were no differences in the self-reported hypoglycaemic or dumping scores assessed by the Sigstad and Edinburgh scales after both conditions. Data are displayed in Figure 3. Peak Sigstad scores were 5.2 and 4.4 for the fat preload and water condition, respectively, *p* = 0.708, 95% CI; −3.9, 5.4) and peak Edinburgh scale scores were 15.6 and 14.1, *p* = 0.260, 95% CI; −1.3, 4.2. Total AUC values were also similar between conditions for both the Sigstad and Edinburgh scale, *p* = 0.567.

## 4. Discussion

This randomised, cross-over study is the first to investigate whether consuming a fat-dominant preload (28 g Brazil nuts), 30 min before a MMTT, could modulate post-MMTT glucose homeostasis in adults without diabetes after SG. Contrary to our hypothesis, the fat preload did not affect nadir plasma glucose concentration or overall postprandial glycaemia (peak and iAUC_0–180 min_) compared with water alone. However, the ingestion of the preload did increase peak postprandial insulin and c-peptide secretion.

In non-surgical individuals, pre-meal fat ingestion has repeatedly been shown to attenuate early postprandial glycaemia by slowing gastric emptying and delaying glucose entry into the circulation [12,21]. In healthy participants, consuming fat (60 g margarine) before a carbohydrate meal reduced both peak and total glucose exposure while significantly delaying gastric emptying [11]. In people with T2D, a preload of 30 mL olive oil markedly slowed gastric emptying and delayed the onset and timing of the postprandial glucose rise; this was accompanied by greater GLP-1 release and a reduced early insulin rise, consistent with a slower rate of glucose appearance in the circulation [10]. More substantial glucose-lowering effects occur when fat is combined with protein: Tricò and colleagues demonstrated that a mixed macronutrient preload (50 g parmesan cheese and 1 boiled egg, 23 g protein, 17 g fat, 2 g carbohydrate) before a 75 g OGTT lowered peak postprandial glucose between 32% and 49% across normal glucose tolerance, impaired glucose tolerance and T2D [12]. Using a dual stable isotope approach, they showed that the attenuated glucose response was driven by enhanced β-cell glucose responsiveness and by a slower rate of oral glucose appearance, particularly in those with impaired glucose tolerance or T2D. This effect was attributed to preload-induced GLP-1 release, which can delay gastric emptying and is reportedly preserved in T2D in a dose-dependent manner. Even very small fat-containing preloads can be effective: in adults with prediabetes or isolated 1-h postprandial hyperglycemia (without T2D), 14.2 g of almond preload reduced 1 h glucose by ~19% and total glucose exposure by ~16% versus water [22]. Taken together, these findings indicate that, in individuals without a history of bariatric surgery, pre-meal fat ingestion alone can blunt early glycaemic excursions primarily by slowing gastric emptying and delaying intestinal glucose delivery driven by GLP-1 release, while the addition of protein further enhances glucose tolerance by augmenting glucose-dependent insulin secretion [6].

The divergent findings observed in our study likely reflect the altered gastrointestinal anatomy and physiology after SG. Removal of the gastric fundus markedly accelerates gastric emptying and nutrient delivery to the small intestine [23], which may reduce the capacity of a fat-dominant preload to further delay gastric transit typically observed in non-surgical individuals. In this context, the fat-dominant preload may have *primed* the gut–pancreas signalling, as indicated by the higher pre-MMTT insulin, c-peptide, and GLP-1 concentration. During the MMTT, higher peak plasma insulin and c-peptide concentration, including numerically higher early post-MMTT insulin and c-peptide responses (iAUC_0–30 min_; medium effect sizes ≈ 0.6), were observed after the fat preload compared with water. These early hormonal increases with the fat-dominant preload may reflect a response to the numerically higher early post-MMTT plasma glucose concentration (iAUC_0–30 min_; medium effect size ≈ 0.6), which in turn could indicate preload-related effects on intestinal nutrient handling during the subsequent meal. An additional contributing factor could be a transient reduction in insulin sensitivity after acute fat intake, as elevated circulating fatty acids can reduce insulin effectiveness [24], a phenomenon that may be accentuated after SG due to rapid lipid delivery and absorption [25]. Thus, while higher post-MMTT peak insulin and c-peptide concentrations were observed, the underlying mechanisms remain uncertain, as the relative contributions of several potential pathways—including gut–pancreas signalling priming, early glycaemic stimulation, and lipid-induced changes in insulin sensitivity—to these hormonal responses cannot be distinguished within the current study design. Despite the observed increase post-MMTT peak insulin and c-peptide levels with the fat preload, nadir and overall postprandial glucose did not differ between conditions. It is important to note that glucagon levels (which play an important role in postprandial glucose regulation) were not measured and changes in glucagon dynamics after the fat-dominant preload could also have contributed to the metabolic pattern observed. Additionally, the relatively modest macronutrient content of the preload in our study (~20 g fat, ~5 g protein) may also have contributed to the absence of overall glycaemic improvement, since studies in non-surgical populations generally report greater efficacy with higher fat and protein doses [6]. Overall, the metabolic response to pre-meal fat after SG likely reflects an interplay of hormonal and gastrointestinal factors rather than a single mechanism.

There is limited evidence on the metabolic effects of virtually carbohydrate-free preloads or fat co-ingestion strategies in individuals who have undergone bariatric surgery. A recent crossover study in post–gastric surgery patients with dumping symptoms but without T2D (including individuals after SG and after subtotal gastrectomy with Roux-en-Y reconstruction) assessed the metabolic impact of adding 15 g of fat directly into a high-carbohydrate liquid meal [26]. Although interpretation is limited by the mixed surgical procedures, the co-ingestion (rather than preload) design, small sample size, and lack of gut hormone or gastric-emptying measurements, the study reported lower glucose concentrations at 60 min and fewer dumping symptoms when fat was incorporated into the meal. This suggests that the timing of fat ingestion may influence postprandial glucose and symptom responses after SG, and that co-ingestion strategies may affect glucose handling differently from preload approaches in this setting. Another relevant comparison comes from preload studies after RYGB, where a fat-only preload (30 mL olive oil) increased significantly both the pre-meal and post-meal plasma GLP-1 concentration but did not increase insulin secretion or improve postprandial glucose compared with water [27]. In our SG cohort, the fat-dominant preload resulted in higher post-MMTT peak insulin and c-peptide concentrations and numerically higher early post-MMTT insulin, c-peptide, and glucose responses (iAUC_0–30 min_; medium effect sizes ≈ 0.6), therefore possibly indicating a different pattern of response to a fat preload after SG compared with RYGB. Moreover, in our study the difference in total plasma GLP-1 concentration post-MMTT between the fat-dominant preload and water was not statistically different. These differences may also reflect preload composition: although Brazil nuts predominantly provide fat, there is a small amount of protein (~5 g), which may have contributed to insulin secretion, whereas the olive oil preload after RYGB contained no protein. Supporting this, a large protein preload (55 g whey) in the same RYGB cohort elicited a stronger metabolic response, lowering early glycaemia and markedly stimulating insulin secretion [27], highlighting the important role of nutrient composition in modulating postprandial physiology after bariatric surgery. Anatomical configuration also diverges: RYGB bypasses the pylorus and duodenum, resulting in more direct stimulation of distal L-cells and exaggerated incretin release, whereas SG preserves pyloric and duodenal continuity and therefore produces a different nutrient-stimulated hormonal profile and glycaemic response [28]. Overall, these observations suggest that the metabolic effects of a fat-dominant preload may differ across bariatric procedures, with our findings in SG indicating a pattern that is not fully aligned with that reported after RYGB, highlighting the need to evaluate preload strategies within each surgical context.

There are limitations to acknowledge. We were unable to directly confirm effects on gastric emptying. Although a paracetamol absorption test was planned, plasma concentrations below 10 mg/L were not reported due to lack of validated in-house precision at this level, resulting in insufficient data to assess gastric emptying and any interpretation regarding effects on gastric emptying should therefore be considered speculative. Moreover, resources did not permit more robust techniques for assessment of gastric emptying such as scintigraphy or stable isotope breath testing as well as the measurement of other entero-pancreatic hormones contributing to glucose homeostasis such as glucose-dependent insulinotropic polypeptide (GIP) and glucagon. We also did not assess glucose absorption, and our static measures of plasma glucose concentration meant that we were not able to distinguish between the appearance and disappearance of plasma glucose. We selected a MMTT because it represents a more physiological nutrient stimulus after SG and carries less risk of provoking dumping symptoms than a liquid OGTT; however, the use of an OGTT may have produced a more pronounced glycaemic and hormonal response, potentially increasing the sensitivity to detect preload effects. The study sample was modest, and participants did not have diagnosed PHH, so these findings may not fully extrapolate to that group. Finally, this proof-of-concept study was powered only for the primary outcome (nadir plasma glucose). Secondary outcomes, including insulin, c-peptide and GLP-1, were analysed as exploratory endpoints, and no adjustment for multiple comparisons was applied. These findings should therefore be interpreted with caution and confirmed in larger, adequately powered studies.

## 5. Conclusions

In conclusion, consuming a standard 28 g portion of Brazil nuts (a virtually carbohydrate-free, fat-dominant preload) 30 min before a MMTT did not alter nadir plasma glucose in post-SG individuals without diabetes or established PHH. Although greater post-MMTT peak insulin and c-peptide concentrations were observed, these were secondary outcomes analysed on an exploratory basis and should be interpreted with caution. These findings apply only to this population and should not be interpreted as evidence against fat-based preloads in individuals with established PHH, in whom responses may differ. The results further suggest that findings from preload studies in non-surgical populations may not be directly translated to individuals after SG. Future studies should incorporate direct measures of gastric emptying and glucose kinetics and explore preload formulations with different fat–protein compositions or alternative timing strategies, particularly in individuals with established PHH.

## Figures and Tables

**Table 1:** Participant characteristics.

	Preload/Water (*n* = 5)	Water/Preload (*n* = 5)	Total (*n* = 10)
Age (years)	45.4 (9.8)	51.6 (9.6)	48.5 (9.7)
Height (cm)	164.2 (3.1)	170.0 (7.2)	167.1 (6.0)
Weight (kg)	97.1 (12.2)	107.3 (29.2)	102.2 (21.8)
Body fat (%)	45.4 (4.7)	43.4 (11.2)	44.4 (8.1)
Systolic blood pressure (mmHg)	109.2 (18.7)	119.8 (16.5)	114.5 (17.5)
Diastolic blood pressure (mmHg)	74.3 (10.8)	69.7 (14.4)	72.0 (12.3)
Resting heart rate (bpm)	67.8 (9.2)	68.1 (11.3)	68.0 (9.7)
BMI (kg/m^2^)	36.1 (5.3)	37.4 (11.1)	38.7 (12.2)
HbA1c (%)	5.3 (0.1)	5.4 (0.4)	5.3 (0.3)
Years since surgery	2.8 (1.3)	3.8 (3.1)	3.4 (2.5)

Data showing participant characteristics by treatment order. Data presented as mean (SD).

**Table 2:** Fasting and postprandial metabolic outcomes.

Outcome	Preload	Water	Difference	*p*-Value	95% CI	Effect Size	*n*
Plasma Glucose							
Nadir plasma glucose (mmol/L)	3.8 (0.7)	3.8 (0.5)	−0.0 (0.5)	0.849	−0.38, 0.32	0.1	10
Fasting plasma glucose (mmol/L)	4.6 (0.5)	4.3 (0.3)	0.3 (0.4)	0.071	−0.03, 0.57	0.6	10
Pre-MMTT (0 min) plasma glucose (mmol/L)	4.3 (0.3)	4.3 (0.2)	0.0 (0.2)	0.691	−0.14, 0.20	0.4	10
Peak plasma glucose (mmol/L)	7.8 (1.4)	7.2 (1.1)	0.6 (1.3)	0.164	−0.30, 1.52	0.5	10
Plasma glucose iAUC_0–30 min_ (mmol∙min/L)	64 (26)	49 (20)	15 (23)	0.080	−31.4, 2.1	0.6	10
Plasma glucose iAUC_0–180 min_ (mmol∙min/L)	135 (117)	127 (85)	8 (97)	0.815	−82.4, 66.7	0.1	9
Plasma Insulin							
Fasting plasma insulin (µU/mL)	9.4 (5.8)	9.2 (5.6)	0.2 (3.0)	0.835	−1.95. 2.35	0.1	10
Pre-MMTT (0 min) plasma insulin (µU/mL)	12.9 (6.7)	8.0 (4.7)	5.0 (2.8)	**<0.001**	2.9, 7.0	0.6	10
Peak plasma insulin (µU/mL)	134 (88)	93 (46)	41 (53)	**0.038**	2.9, 79.1	0.6	10
Plasma insulin iAUC_0–30 min_ (µU∙min/mL)	2200 (1617)	1596 (941)	605 (958)	0.077	−1290, 81	0.6	10
Plasma insulin iAUC_0–180 min_ (µU∙min/mL)	5036 (2883)	4560 (2203)	476 (1788)	0.422	−225, 2665	0.3	9
Plasma C-peptide							
Fasting plasma C-peptide (pg/mL)	1057 (376)	974 (312)	84 (150)	0.111	−23.4, 190.8	0.3	10
Pre-MMTT (0 min) plasma C-peptide (pg/mL)	1226 (382)	957 (337)	267 (99)	**<0.001**	198, 340	0.6	10
Peak plasma C-peptide (pg/mL)	5542 (3049)	4332 (1797)	1210 (1667)	**0.040**	17.3, 2402.7	0.5	10
Plasma C-peptide iAUC_0–30 min_ (pg∙min/mL)	75,920 (56,611)	59,850 (35,782)	16,071 (28,837)	0.112	−36,700, 4558	0.6	10
Plasma C-peptide iAUC_0–180 min_ (pg∙min/mL)	241,919 (132,678)	243,157 (93,450)	1238 (89,798)	0.968	−67,787, 70,263	0.1	9
Plasma total GLP-1							
Fasting plasma total GLP-1 (pmol/L)	23.2 (8.8)	23.7 (10.4)	−0.5 (10.0)	0.876	−7.7, 6.7	0.1	10
Pre-MMTT (0 min) plasma total GLP-1 (pmol/L)	37.9 (9.8)	25.7 (10.3)	12.2 (14.8)	**0.028**	1.6, 22.8	0.5	10
Peak plasma total GLP-1 (pmol/L) *	114.4 (98.6)	94.8 (58.4)	19.1 (31.6)	0.232	−17.4, 161.5	0.5	10
Plasma total GLP-1 iAUC_0–30 min_ (pmol∙min/L)	2016 (1656)	1523 (778)	493 (1778)	0.403	−1765, 779	0.3	10
Plasma total GLP-1 iAUC_0–180 min_ (pmol∙min/L)	5069 (6071)	3754 (2452)	1315 (6867)	0.581	−6594, 3963	0.2	9

Data represent mean (SD). Data analysed using paired *t*-tests. * Non-parametric Wilcoxon signed-rank test applied and medians are presented. *p*-values in bold represent statistically significant difference between preload and water (*p* < 0.05). Data are *n* = 9 due to the development of hypoglycaemia for one participant at 60 min and resulting cessation of experiment.

## Data Availability

The data described in this article are not publicly available due to ethical and privacy considerations. Data are available from the corresponding author upon reasonable request.

## Supplementary Materials

The following supporting information can be downloaded at: https://www.mdpi.com/article/10.3390/nu18030469/s1.
