# Supplementary material for: The Impact of a Fat-Dominant Preload Before a Carbohydrate-Rich Meal on Glucose Homeostasis in Patients Without Diabetes After Sleeve Gastrectomy: A Proof-of-Concept, Randomised, Open-Label, Crossover Study
Source: Nutrients. 2026 Jan 31;18(3):469. doi: 10.3390/nu18030469 (PMC12899494; doi:10.3390/nu18030469)
Supplement: Supplementary file 1 [file nutrients-18-00469-s001.zip › nutrients-4109160-supplementary.pdf]

Supplementary Material

Inclusion Criteria

1. Aged ≥18 years old
2. Subjects ≥1 year after sleeve gastrectomy (SG)
3. Able to understand written and spoken English
4. Able to give informed consent
5. Happy for their GP to be notified of their study participation

Exclusion criteria:

1. Use of any glucose-lowering medication (including insulin)
2. Adrenal insufficiency and/or substitution with glucocorticoids
3. eGFR ≤ 45 ml/min/1.73m2
4. weight ≤ 50kg
5. Recent active infection (an active infection will be any infection over the last 10 days)
6. Current use or history of treatment within 6 weeks with systemic glucocorticoids (oral or injectable) not including use of topical (e.g. eye drops or topical creams) or inhaled glucocorticoids
7. People with allergy or severe intolerance to the mixed meal tolerance test as assessed by the clinician (e.g., severe milk protein allergy, lactose and gluten intolerance)
8. People with allergy or intolerance to paracetamol or to nuts
9. Other bariatric procedure except of SG
10. Previous major revisional bariatric surgery (except of previous gastric band which has been removed)
11. Hb <100 g/L at screening blood tests
12. HbA1C ≥6.5% or ≥48 mmol/L at screening blood tests
13. Currently pregnant or breastfeeding
14. Diagnosis of Type 1 Diabetes
15. Current diagnosis of Type 2 Diabetes (defined as HbA1C ≥6.5%/ ≥48 mmol/L at screening blood tests or HbA1C <6.5%/ <48 mmol/l at screening blood tests but on glucose lowering medications over last 3 months)
16. Patients with diagnosis of Epilepsy

17. Participating in another Clinical Trials of Investigational Medicinal Products (CTIMP) study within <1 month of screening

18. Having a formal previous diagnosis of postprandial hypoglycaemia

19. Currently on Metoclopramide, domperidone or colestyramine as they can affect paracetamol absorption as per SPC (Summary of Product Characteristics) for paracetamol.

20. Currently on acarbose, diazoxide, octreotide or other treatment for postprandial hypoglycaemia

21. Any concurrent condition, in the judgment of investigator and/ or GP practitioner, that could interfere with the safety and study conduct or interpretation of study results
